# A Staged Hybrid Technique for Large Benign Colorectal Polyps with Severe Fibrosis: A Retrospective Cohort Study

**DOI:** 10.1055/a-2926-6624

**Published:** 2026-08-12

**Authors:** Robert Eckersley, Adam Humphries, Brian Saunders

**Affiliations:** 1 Wolfson Unit for Endoscopy 105692 St Mark’s Hospital and Academic InstituteLondonLondonUnited Kingdom of Great Britain and Northern Ireland; 2 Department of Surgery and Cancer 4615 Imperial College LondonLondonUnited Kingdom of Great Britain and Northern Ireland

**Keywords:** endoscopy lower GI tract, polyps/adenomas/..., endoscopic resection (polypectomy, ESD, EMRc, ...)

## Abstract

**Background and aims** Severe submucosal fibrosis presents a major challenge during endoscopic resection (ER) of large colorectal polyps, often precluding standard endoscopic mucosal resection (EMR) or endoscopic submucosal dissection (ESD). This challenge is particularly relevant in elderly or comorbid patients when procedural safety is paramount. We describe the safety and efficacy of the novel Polyp Excision with Thermal Ablation and avuLsion (PETAL) technique, a staged hybrid approach combining wide-field EMR, thermal ablation, and avulsion.

**Methods** This retrospective cohort study included consecutive patients undergoing PETAL for benign-appearing colorectal polyps ≥20 mm with severe fibrosis between January 2015 and December 2024. Primary outcomes included adverse events and recurrence at follow-up. Secondary outcomes included eradication rates and the need for surgery.

**Results** Sixty-nine patients were included (median age, 71 years; 38% ASA III-IV; 20% on antithrombotics). The median lesion size was 50 mm, with 13.0% recurrent lesions. Delayed bleeding occurred in 2.9%, with no perforations or deep mural injuries. Follow-up data were available for 61 patients (88%), with median duration 40 months. Recurrence at first follow-up was 45.9%, but typically small and amenable to repeat endoscopic therapy. Cumulative eradication rates were 91.1% and 98.2% at the second and third follow-ups, respectively. One patient required surgery.

**Conclusions** PETAL is a safe and effective strategy for large colorectal polyps with severe fibrosis, achieving high rates of eventual eradication despite frequent early recurrence. Its staged, low-risk profile makes it particularly suitable for elderly or comorbid patients and may represent an alternative to higher-risk techniques such as ESD and full-thickness resection.

## Introduction

Endoscopic resection (ER) is the preferred treatment for large non-pedunculated colorectal polyps, avoiding the morbidity and mortality associated with surgical resection. 1 Conventional techniques such as endoscopic mucosal resection (EMR) are effective for most lesions; however, severe submucosal fibrosis can render these techniques ineffective by eliminating the submucosal lifting plane. 2 3

Advanced resection strategies have been developed to address this challenge. Endoscopic submucosal dissection (ESD) allows en bloc resection but is technically demanding, time-consuming, and associated with increased risks, particularly in Western practice. 4 5 6 Endoscopic intermuscular dissection (EID) has emerged as a modification for fibrotic low rectal lesions but requires advanced expertise and carries significant risk of perforation. 7 Full-thickness resection devices (FTRD) provide another option for non-lifting lesions, although their use is limited by lesion size, anatomical constraints, and potential complications. 8

These challenges are amplified in elderly or comorbid patients, where prolonged procedures and complications may have significant clinical consequences. 9 In such cases, a staged, lower-risk endoscopic approach may be preferable to aggressive resection or surgery.

Current guidance from the European Society of Gastrointestinal Endoscopy emphasises complete endoscopic resection where feasible. 1 However, there is limited specific guidance for the management of severely fibrotic non-lifting lesions. The novel Polyp Excision with Thermal Ablation and avuLsion (PETAL) technique was developed as a pragmatic hybrid approach combining wide-field EMR with targeted ablation and mechanical avulsion of fibrotic tissue. To our knowledge, this is the first study describing a structured staged resection strategy for severely fibrotic benign colorectal lesions. This study evaluates the safety and efficacy of PETAL, with particular emphasis on its role in higher-risk patient populations.

## Methods

### Study Design and Setting

This retrospective cohort study was conducted at a tertiary referral centre. Consecutive patients undergoing ER using the PETAL technique for benign-appearing colorectal polyps >20 mm with severe fibrosis between January 2015 and December 2024 were reviewed. Severe fibrosis was defined by the presence of a non-lifting sign following submucosal injection and/or failure to identify a submucosal plane during attempted resection, typically in the context of a large dominant nodule or previous endoscopic manipulation. Although a formal fibrosis grading system was rarely used, these appearances are consistent with Matsumoto Grade F2. 10 Lesions with diffuse fibrosis throughout the entire lesion were not considered ideal candidates. A substantial proportion of patients were elderly and/or had significant comorbidities, influencing the decision to pursue a lower-risk endoscopic strategy over surgery or advanced dissection techniques.

Primary outcomes included adverse events, specifically delayed bleeding, deep mural injury and perforation, as well as recurrence at follow-up. Secondary outcomes included endoscopic eradication and the requirement for surgical intervention.

Patients underwent surveillance in accordance with international guidelines, with first surveillance endoscopy at 3–6 months. If residual or recurrent adenoma was identified, repeat endoscopic treatment was performed and further surveillance was scheduled according to endoscopist judgement, usually within 3–6 months. If no recurrence was seen, subsequent surveillance followed standard post-EMR surveillance pathways. Recurrence was defined as the presence of visible adenomatous tissue at the resection site on surveillance endoscopy. Complete eradication was defined as the absence of visible residual or recurrent adenoma at the resection scar. Routine biopsy of normal-appearing scar tissue was not mandated.

Eradication rates at each surveillance timepoint were calculated using patients who had undergone that surveillance examination as the denominator. Patients who had not yet reached the surveillance interval, declined further follow-up, or were lost to follow-up were excluded from the analysis for that timepoint.

Ethical approval was waived due to the retrospective nature of the study, which was conducted in accordance with the Declaration of Helsinki.

### PETAL procedure

All procedures were performed as day-case procedures under either endoscopist-delivered conscious sedation, or anaesthetist-delivered propofol deep sedation. Procedures were performed by two expert therapeutic endoscopists or supervised trainees.

Single channel adult or paediatric colonoscopes were used. Short straight cap distal attachments were commonly used. Polyp size was measured endoscopically, with the open biopsy forceps for reference (diameter 7 mm). Lesions were selected for PETAL following careful optical assessment with high-definition white light imaging, narrow-band-imaging and magnification where available. Lesions with endoscopic features of submucosal invasion, including NICE 3 or JNET2B/3 features, ulceration, depressed morphology, marked surface disruption, convergent folds, or a firm invasive-appearing non-lifting component, were excluded from PETAL and referred for alternative management. 11 12

**Fig. 1:**
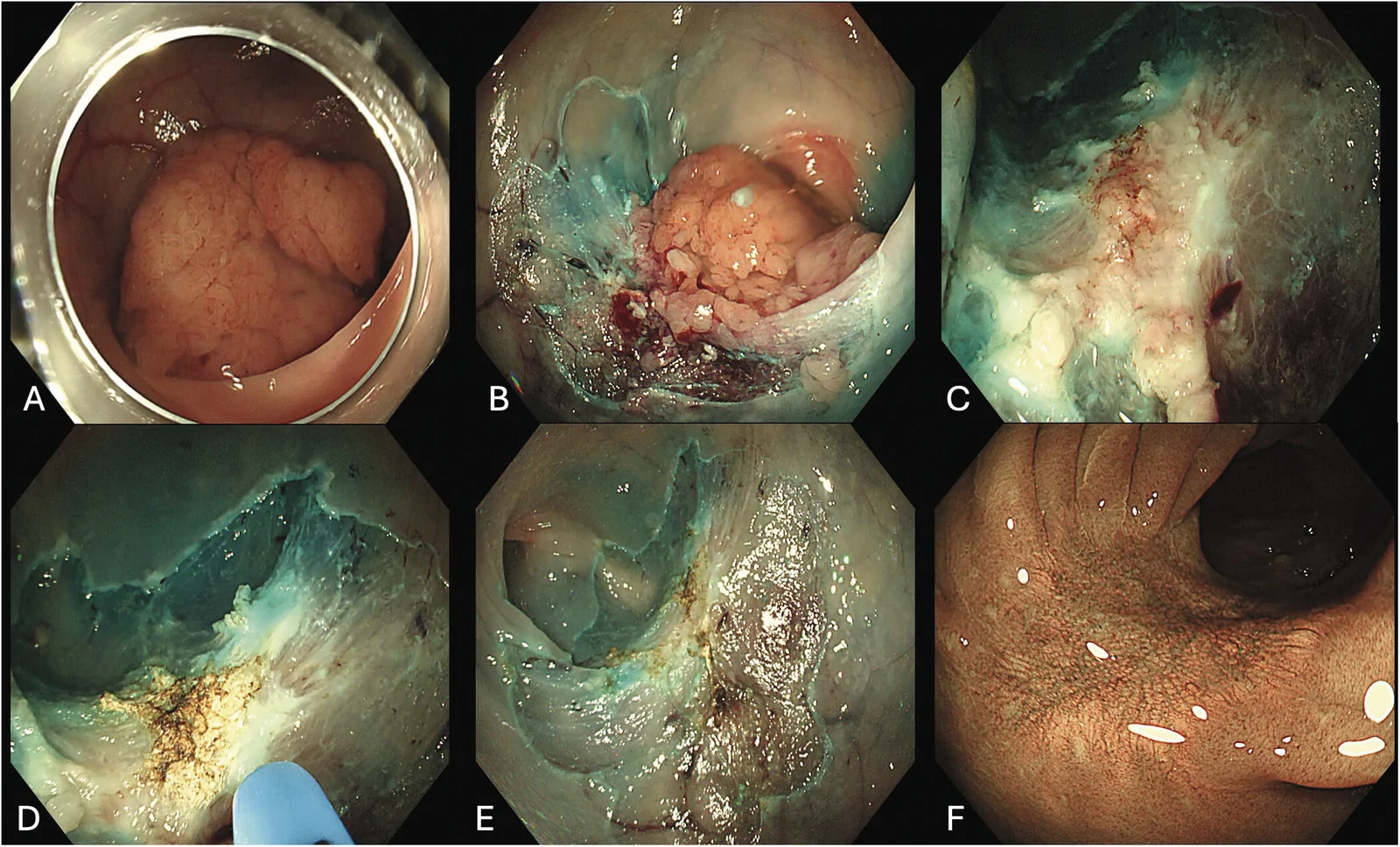
PETAL procedure: ( **A** ) Large benign colorectal polyp; ( **B** ) Peripheral EMR; ( **C** ) Severe central fibrosis prohibiting conventional snare or knife resection; ( **D** ) APC ablation of central fibrosis; ( **E** ) Scar cleaning with cold forceps avulsion and repeat APC ablation (ACA); and ( **F** ) Complete resolution at follow-up (NBI).

The PETAL procedure ( Fig. 1 ) first involves circumferential, wide-field EMR of the non-fibrotic polyp component to isolate and expose the fibrotic central base, creating an appearance similar to the petals around the head of a flower (image C, Fig. 1 ). Submucosal injection with a mixed solution of 95 mL 0.9% sodium chloride, 5 mL adrenaline (1:10,000), and 0.5 mL methylene blue was used. A variety of electrosurgical snares were used, with sizes ranging from 10 to 20 mm depending on operator preference. All resections were performed using an ERBE VIO 2 or 3 electrosurgical generator (ERBE Elektomedezin GmbH, Tübingen, Germany) in coagulation mode (VIO2: forcedCOAG 60 W; VIO 3: forcedCOAG 4.5). Once the central fibrotic core was isolated, it was widely fulgurated with APC (VIO 2: forcedAPC 40 W, flow 0.8 L/min; VIO 3: forcedAPC 2.5–3.5, flow 0.8 L/min). APC was intentionally applied first to devitalise the fibrotic adenomatous tissue, allowing safer mechanical avulsion with biopsy forceps before repeat APC ablation of any residual tissue beneath. Without prior application of APC, the fibrotic tissue was often too dense to be removed safely with forceps alone.

Snare-tip soft coagulation of the EMR was not routinely performed during the study period. This reflects the evolution of practice over the cohort period and is now recognised as a limitation.

Due to large, fibrotic defects and the expected high recurrence rates at first surveillance, clip closure was not routinely performed.

### Statistical Analysis

Continuous variables were summarised using medians with interquartile ranges, and compared using the Mann–Whitney *U* test. Categorical variables were summarised as frequencies and percentages, and compared using the chi-squared test.

Exploratory analyses of factors associated with recurrence at first surveillance colonoscopy were performed using Firth penalised logistic regression, with lesion size (per 10 mm), right-sided location and previous manipulation/recurrence chosen a priori. Odds ratios with 95% confidence intervals are reported.

Statistical significance was defined as a two-sided p-value of less than 0.05. All statistical analyses were performed using R (version 4.5.0; R Foundation for Statistical Computing, Vienna, Austria).

## Results

Sixty-nine PETAL procedures were identified. Patient and polyp characteristics are summarised in Table 1 . Then, 38/69 (55.1%) were male. Median age was 71 years (IQR 63–80), 26/69 (37.7%) had severe medical co-morbidity with ASA grade III-IV, and 14/69 (20.3%) were on antithrombotic therapy.

**Table 1:** Patient and polyp characteristics.

	Resections ( *n* = 69)
Patient demographics	
Sex (%)	
Male	38 (55.1)
Female	31 (44.9)
Age (years)	
Median (IQR)	71 (63-80)
ASA (%)	
I to II	43 (62.3)
III to IV	26 (37.7)
Antithrombotic therapy (%)	
Yes	14 (20.3)
No	55 (79.7)
**Polyp characteristics**	
Site (%)	
Right colon	32 (46.4)
Left colon	21 (30.4)
Rectum	16 (23.2)
Lesion diameter (mm)	
Median (IQR)	50 (37.5-70)
Morphology—Paris classification (%)	
0-Ip	0 (0)
0-Isp	2 (2.9)
0-Is	36 (52.2)
0-Is+IIa	13 (18.8)
0-IIa	18 (26.1)
0-IIb	0 (0)
0-IIc	0 (0)
0-III	0 (0)
**Previous manipulation**	
Biopsy (%)	
Yes	2 (2.9)
No	67 (9.7)
Tattoo (%)	
Yes	0 (0)
No	69 (100)
Incomplete ER attempt (%)	
Yes	4 (5.8)
No	65 (94.2)
Recurrent lesions (%)	
Yes	9 (13.0)
No	60 (87.0)

Then, 32 (46.4%) lesions were located in the right colon, 21 (30.4%) in the left colon, and 16 (23.2%) in the rectum. Median lesion diameter was 50 mm (IQR 37.5–70 mm) with 51/69 (73.9%) protruding (Paris 0-Isp, 0-Is, or 0-Is+IIa) and 18/69 (26.1%) flat lesions (Paris 0-IIa).

Then, 6/69 (8.7%) lesions had been previously manipulated with either prior biopsy or incomplete ER attempt and 9/69 (13.0%) were recurrent lesions.

Outcomes are summarised in Table 2 . No resection defects were closed with through-the-scope clips or other closure devices. Delayed bleeding requiring re-admission to hospital occurred in two cases (2.9%). Both bleeding events occurred following recommencement of antithrombotic therapy. Both required repeat therapeutic endoscopy without blood transfusion. There were no incidences of deep mural injury, perforation, or post-polypectomy syndrome. There were no procedure-related mortalities. Histopathology showed low-grade dysplasia in 32 cases (46.4%), high-grade-dysplasia in 36 (52.2%), and adenocarcinoma in one case (1.4%).

**Table 2:** Procedure outcomes.

	Resections ( *n* = 69)
Adverse events	
Delayed bleeding (%)	
Yes	2 (2.9)
No	67 (97.1)
Deep mural injury (%)	
Yes	0 (0)
No	69 (100)
Perforation (%)	
Yes	0 (0)
No	69 (100)
Post-polypectomy syndrome (%)	
Yes	0 (0)
No	69 (100)
**Histopathology**	
Dysplasia (%)	
Low-grade	32 (46.4)
High-grade	36 (52.2)
Adenocarcinoma	1 (1.4)

**Fig. 2:**
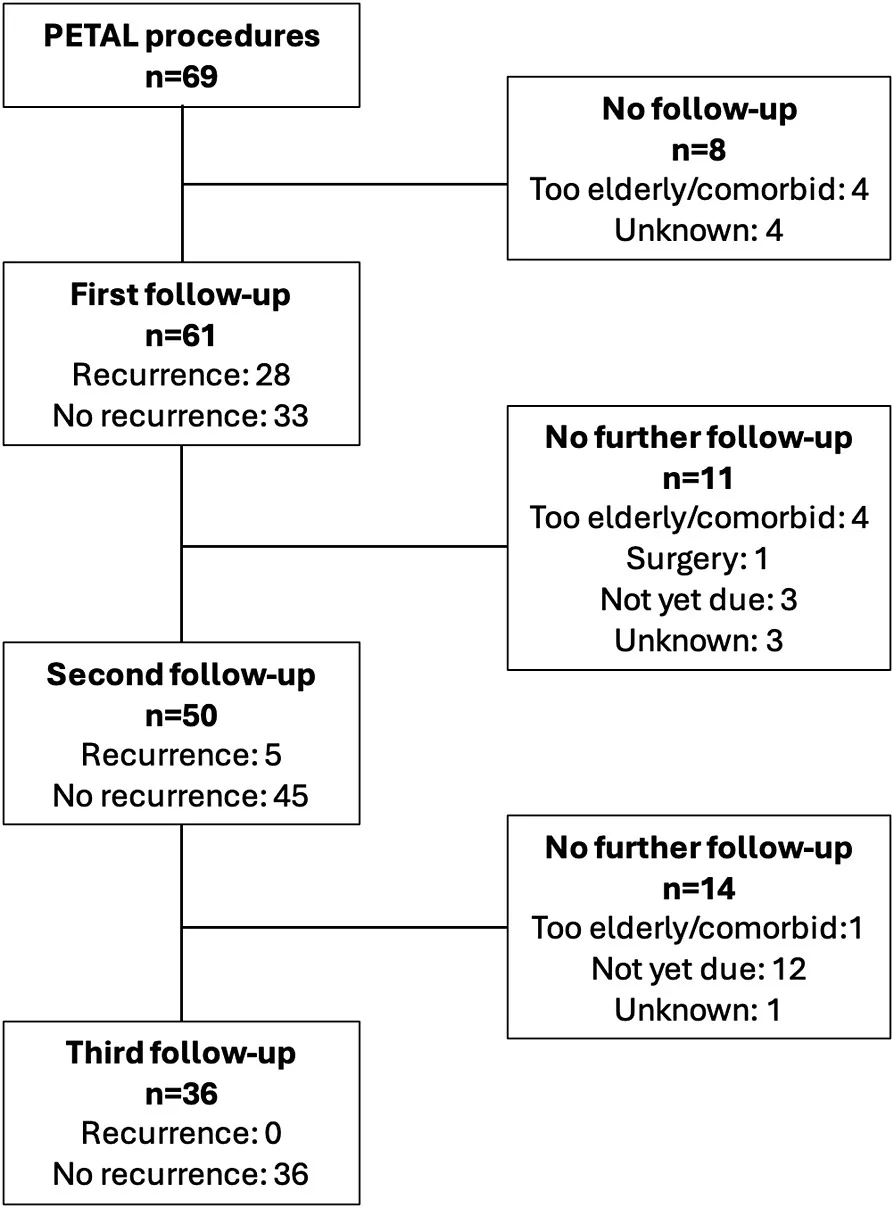
Follow-up and recurrence flowchart.

Follow-up data were available for 61 patients (88.4%), with median follow-up duration 40 months ( Fig. 2 ). Recurrence and eradication rates are summarised in Table 3 . At first surveillance colonoscopy, recurrence was identified in 28/61 patients (45.9%), but cumulative eradication improved to 91.1% and 98.2% at the second and third surveillance colonoscopies, respectively. Recurrence was generally small and endoscopically manageable, with median size 8 mm (IQR 5–15 mm) and 10 mm (IQR 5–17.5 mm) and the first and second follow-ups, respectively. Treatment of recurrence included cold snare or underwater EMR, with repeat APC ablation and avulsion for any fibrotic scar recurrence. Histology from treated recurrence was available in all cases, with 22 low-grade dysplasia and five high-grade dysplasia. One additional patient developed recurrent high-grade dysplasia at 3-month surveillance but was referred for surgery because the endoscopist suspected underlying invasive malignancy. The one patient with adenocarcinoma at index resection was found to have a T1b, moderately differentiated adenocarcinoma with lymphovascular invasion. Following MDT discussion, the patient opted for surveillance, with no recurrence seen at 18 months’ follow-up.

**Table 3:** Recurrence and eradication rates.

	*n*
Recurrence	
First follow-up (%)	
Yes	28 (45.9)
No	33 (54.1)
Second follow-up (%)	
Yes	5 (10.0)
No	45 (90.0)
Third follow-up (%)	
Yes	0 (0.0)
No	36 (100.0)
**Cumulative eradication**	
First follow-up (%)	
Yes	33 (54.1)
No	28 (45.9)
Second follow-up (%)	
Yes	51 (91.1)
No	5 (8.9)
Third follow-up (%)	
Yes	55 (98.2)
No	1 (1.8)

Lesions with recurrence at first surveillance colonoscopy were significantly larger than those without recurrence (median 65 mm [IQR 40–80] vs. 50 mm [IQR 35–60], *p* = 0.020). Lesion location and previous manipulation/recurrence were not associated with recurrence at first surveillance. Exploratory Firth penalised logistic regression suggested that lesion size, right-sided location and previous manipulation may be associated with recurrence at first surveillance ( Table 4 ).

**Table 4:** Factors associated with recurrence at first surveillance colonoscopy. Odds ratios estimated using Firth penalised logistic regression.

Variable	Univariable OR (95% CI)	*p* -Value	Multivariable OR (95% CI)	*p* -Value
Lesion size per 10 mm	1.32 (1.04–1.75)	0.019	1.70 (1.22–2.54)	0.001
Right colon vs. left colon/rectum	1.22 (0.45–3.32)	0.697	3.74 (1.07–16.00)	0.039
Previously manipulated/recurrent vs. naïve	1.75 (0.55–5.86)	0.344	4.72 (1.20–21.58)	0.025

## Discussion

This study demonstrates that the PETAL technique is a safe and effective approach for the management of large colorectal polyps with severe submucosal fibrosis. In a cohort of 69 patients, characterised by a median age of 71 years and a substantial proportion with high ASA grades and/or antithrombotic therapy, PETAL demonstrated a favourable safety profile, with only two cases of delayed bleeding and no perforations or deep mural injury. Recurrence at the first follow-up was 45.9%, substantially higher than expected after standard EMR of non-fibrotic lesions. However, recurrence was frequently small, and reflects the intrinsic difficulty of treating fibrotic lesions and the staged nature of the PETAL strategy, rather than procedural failure. With repeat endoscopic therapy, high rates of eventual eradication rates of 98% were achieved. These findings support its role as a potential low-risk intervention. PETAL therefore represents a shift from single-session radical resection towards staged eradication, with patients counselled that repeat endoscopic treatment is commonly required. T may be more appropriate in elderly or comorbid patients where procedural risk outweighs the benefits of aggressive resection.

Severe fibrosis remains one of the most significant technical challenges in advanced endoscopic resection. Conventional EMR is frequently ineffective due to non-lifting, while ESD, although capable of achieving en bloc resection, becomes considerably more complex and hazardous in this setting. 12 Fibrosis is associated with prolonged procedural time, loss of dissection planes, and increased risk of perforation, particularly in Western practice where procedural volumes and experience may be more variable. 13 14 These risks are especially relevant in elderly or comorbid patients, in whom complications may result in substantial morbidity and prolonged hospitalisation. In contrast, the PETAL technique avoids deep submucosal dissection and instead adopts a staged eradication strategy that prioritises procedural safety.

Several endoscopic resection techniques have been described for fibrotic or non-lifting polyps. Hot avulsion and cold forceps avulsion with adjunctive snare-tip soft coagulation techniques use mechanical avulsion with adjunctive thermal therapy to treat non-snareable residual adenoma and have demonstrated the feasibility of scar-based eradication strategies. 15 16 Underwater EMR has emerged as an alternative strategy for recurrent and fibrotic lesions, reducing dependence on submucosal lifting, and facilitating snare capture of non-lifting tissue. 17 However, severe fibrosis may still limit complete resection in some cases, and PETAL may provide an alternative approach when a substantial fibrotic core remains resistant to snare-based techniques. EID has also been proposed as an alternative approach for fibrotic rectal lesions, allowing dissection within deeper tissue planes. While promising, this technique is technically demanding, requires specialist expertise, and carries a significant risk of perforation. 7 Its applicability is therefore limited to selected centres and operators. Similarly, FTRD provide an option for non-lifting lesions, enabling en bloc resection; however, their use is constrained by lesion size, anatomical considerations, and potential complications such as perforation or appendicitis. 8 In addition, FTRD is typically restricted to smaller lesions, whereas the PETAL technique in this series was successfully applied to lesions up to 85 mm in size.

From a clinical perspective, the PETAL technique addresses an important therapeutic gap. It may provide a pragmatic and broadly applicable option for lesions that are unsuitable for conventional or underwater EMR and high-risk for ESD or EID, while avoiding the limitations of FTRD. This is particularly relevant in elderly patients and those with significant comorbidities, where surgical resection or prolonged advanced endoscopic procedures may carry unacceptable risk. In such patients, a safe, repeatable, and outpatient-based approach is highly advantageous. An important limitation of PETAL is that ablation and avulsion of the fibrotic core precludes complete histopathological assessment of all treated tissue. Consequently, occult invasive disease within the fibrotic component cannot be entirely excluded despite careful optical assessment. For this reason, accurate optical diagnosis and lesion selection are essential, and lesions with features suggestive of deep submucosal invasion should not be considered for PETAL.

Exploratory multivariable analysis identified several factors independently associated with recurrence at first surveillance. Increasing lesion size, right-sided location, and previous manipulation or recurrence were all associated with a higher likelihood of recurrence at first surveillance colonoscopy. However, given the modest sample size and exploratory nature of the analysis, these observations should be interpreted cautiously and require prospective validation in larger cohorts.

This study has several limitations. The retrospective design introduces potential selection and reporting biases, and the relatively small sample size limits generalisability. Furthermore, decreasing patient numbers at later follow-up may have influenced eradication estimates. The absence of a comparator group precludes direct comparison with alternative techniques such as ESD or FTRD, and the total number of fibrotic lesions managed using alternative techniques during the study period was not prospectively recorded. The assessment of fibrosis did not routinely use a validated fibrosis grading system, but was instead often based on endoscopic impression, and may be subject to operator variability. The size of the fibrotic component was not recorded systematically, preventing analysis of whether fibrotic burden influenced recurrence. Finally, formal measures of frailty and comorbidity were not systematically recorded, although these factors clearly influenced clinical decision-making.

Despite these limitations, this study reflects real-world practice in a cohort of patients with challenging lesions and elevated procedural risk. The findings suggest that PETAL may represent a safe, effective, and pragmatic strategy for managing fibrotic colorectal polyps, particularly in elderly or comorbid patients where minimising risk is paramount. Further prospective and comparative studies are warranted to better define its role relative to other advanced endoscopic techniques.
